# The combination of angiogenesis and blood vessel invasion as a prognostic indicator in primary breast cancer

**DOI:** 10.1038/sj.bjc.6600921

**Published:** 2003-06-10

**Authors:** T Kato, S Kameoka, T Kimura, T Nishikawa, M Kobayashi

**Affiliations:** 1 Department of Surgery II, School of Medicine, Tokyo Women's Medical University, 8-1 Kawadacho, Shinjuku-ku, Tokyo 162-8666, Japan; 2 Department of Surgical Pathology, School of Medicine, Tokyo Women's Medical University, 8-1 Kawadacho, Shinjuku-ku, Tokyo 162-8666, Japan; 3 Department of Pathology, School of Medicine, Tokyo Women's Medical University, 8-1 Kawadacho, Shinjuku-ku, Tokyo 162-8666, Japan

**Keywords:** angiogenesis, blood vessel invasion, breast cancer, haematogenous dissemination, long-term survival, microvessel density

## Abstract

This study was undertaken to examine the interaction between the combination of angiogenesis and blood vessel invasion (BVI) and haematogenous metastasis, and to determine the prognostic significance of that combination in predicting 20-year relapse-free survival (RFS) and overall survival (OS) rates in primary breast cancer. Five hundred and nine patients were studied. We investigated 11 factors, including average microvessel count (AMC)/BVI, lymph-node status (*n*), clinical tumour size (*T*), histological grade (HG), lymphatic vessel invasion (LVI), p53, proliferating cell nuclear antigen (PCNA), c-*erb*B-2, mitotic index (MI), apoptotic index, and tumour necrosis (TN). Blood vessel invasion was detected by both factor VIII-related antigen and elastica van Gieson staining. To evaluate the best objective method to quantify microvessel density in angiogenesis, AMC was employed. The rate of AMC-high and BVI-positive tumours was 32.6 and 29.3%, respectively. That of both AMC-high and BVI-positive tumours was 10.1%. Univariate analysis showed that AMC/BVI, *n*, *T*, HG, LVI, p53, PCNA, MI, and TN were significantly predictive of RFS and OS. By multivariate analysis, AMC/BVI was the strongest independent prognostic factor for 20-year RFS (relative risk (RR)=5.5; *P*<0.0001) and for 20-year OS (RR=4.3; *P*<0.0001). Lymph-node status was still considered a powerful prognostic indicator; however, the combination of AMC and BVI provided more reliable prognostic information than lymph-node status for haematogenous dissemination.

There have been many reports about the prognostic factors in breast cancer of which lymph-node status remains a major conventional prognostic factor and has been clinically used as a measure for disseminating the disease, while some investigators have reported that lymph-node metastases have not necessarily been associated with haematogenous dissemination (Fisher *et al*, 1981; Menard *et al*, 1994; Braun *et al*, 2000). Moreover, several additional biologic markers, such as angiogenesis, p53, proliferating cell nuclear antigen (PCNA), c-*erb*B-2, apoptosis, mitosis, and necrosis have been related to tumour proliferation and development, but their effects on tumour haematogenous metastasis are by no means clear, and there are still controversies regarding the best possible prognostic index for breast cancer patients (Thor *et al*, 1992; Gilchrist *et al*, 1993; Zhang *et al*, 1998; Kato *et al*, 1999; Beenken *et al*, 2001; Sirvent *et al*, 2001).

Some studies have suggested that microvessel density indicated representative angiogenesis of the tumour and was an independent and highly significant prognostic factor for both node-negative and node-positive patients (Weidner *et al*, 1991; Gasparini *et al*, 1994; Simpson *et al*, 1996; Heimann *et al*, 1996; Kato *et al*, 2001). However, other authors reported that angiogenesis did not predict recurrence in patients with primary breast cancer (Van Hoef *et al*, 1993; Goulding *et al*, 1995). The significance of angiogenesis remains controversial because of studies that varied in patient selection, length of time of patient follow-up, antibody used to detect endothelial cells, sample size, method of counting microvessels, and the patients' race.

Recent reports have suggested that peritumour lymphatic and blood vessel invasion (BVI) (vascular invasion) (Lee *et al*, 1990; Mansi *et al*, 1991; Fox *et al*, 1997) or peritumour lymphatic vessel invasion (LVI) (Clemente *et al*, 1992; Gasparini *et al*, 1994; Lauria *et al*, 1996) are significant prognostic factors. However, there are a few recent studies of the prognostic significance of peritumour BVI (Gasparini *et al*, 1994; Lauria *et al*, 1996; Kato *et al*, 2000,^2002^). It has been more difficult to identify invasion of small vessels and capillaries and in the larger vessels surrounded by elastic tissue in breast cancer in routine haematoxylin and eosin (H&E)-stained tissue sections than in gastric or colorectal cancer. A wide range of frequencies has been reported for BVI among patients with breast cancer and the prognostic significance of BVI has not yet been made clear (Weigand *et al*, 1982; van de Velde *et al*, 1986; Gasparini *et al*, 1994; Lauria *et al*, 1996; Kato *et al*, 2000, 2002).

Tumours that have acquired the ability of high neovascularisation are likely to involve blood vessels. However, all tumours with high neovascularisation do not have the ability to invade vessels. Gould *et al* (1983) reported that bronchial carcinoid tumours are highly vascular, but they do not spread beyond the lung. Angiogenesis is necessary for a tumour to grow but not sufficient for it to metastasise. Thus, there are definitely biological differences between the ability of tumours to form high neovascularisation and their ability to invade blood vessels. And both the growth of tumours dependent on angiogenesis and the tumours spreading beyond the primary site are dependent on access to the vasculature and are important for haematogenous metastasis.

This study was undertaken to examine the interaction between the combination of angiogenesis and BVI and haematogenous dissemination, and to determine the absolute and relative values of that combination using both Factor VIII-related antigen and elastica van Gieson staining, as we detected by our method published previously (Kato *et al*, 2001,^2002^). Moreover, the clinicopathological significance of p53, PCNA, c-*erb*B-2, and apoptosis, relatively new prognostic factors, and conventional prognostic factors, especially lymph-node status was evaluated in predicting relapse-free survival (RFS) and overall survival (OS) rates with a long-term follow-up in Japanese patients with breast cancer.

## PATIENTS AND METHODS

### Patients

Data for this study were collected from 509 breast cancer patients selected from those operated on between 1971 and 1990 at Tokyo Women's Medical University Hospital for whom we had sufficient clinical and pathologic material to determine all the biological markers. The distribution of the main clinicopathologic data for the entire 509-patient population is given in Table 1

**Table 1:** Clinicopathologic characteristics of 509 patients

**Characteristics**	**No. of patients (%)**
Patients enrolled	509
Age (years)	
Median	49
Range	15–84
Survival follow-up (years)	
Median	9
Range	1–20
Recurrence	103 (20.2)
Deaths	83 (16.3)
Menopausal status	
Pre	256 (50.3)
Post	228 (44.8)
Unknown	25 (4.9)
Clinical tumour size (*T*)	
T1	194 (38.1)
T2	251 (49.3)
T3	64 (12.6)
Lymph-node status	
Negative	307 (60.3)
Positive	202 (39.7)
Average microvessel count	
Low	327 (67.4)
High	158 (32.6)
Blood vessel invasion	
Negative	357 (70.7)
Positive	148 (29.3)
AMC/BVI	
Low/negative	235 (48.7)
High/negative	105 (21.7)
Low/positive	94 (19.5)
High /positive	49 (10.1)
Lymphatic vessel invasion	
Negative	387 (76.8)
Positive	117 (23.2)
Histological grade	
HG I (Well)	219 (43.5)
HG II (Moderate)	141 (28.0)
HG III (Poor)	144 (28.5)
p53	
Negative	334 (69.7)
Positive	145 (30.3)
c-*erb*B-2	
Negative	305 (65.3)
Positive	162 (34.7)
PCNA	
Negative	258 (53.3)
Positive	226 (46.7)
MI	
Negative	327 (65.1)
Positive	175 (34.9)
AI	
Negative	397 (79.1)
Positive	105 (20.9)
Tumor necrosis	
None	309 (61.6)
Central	76 (15.1)
Comedo	117 (23.3)
Histological classification	
Infiltrating ductal carcinoma	466 (92.4)
Infiltrating lobular carcinoma	20 (4.0)
Others	18 (3.6)

PCNA=proliferating cell nuclear antigen; MI=mitotic index; AI=apoptotic index.

. Two hundred and sixty-four of these patients (51.9%) were treated with radical mastectomy, 153 patients (30.0%) with modified radical mastectomy, and 92 patients (18.1%) with extended radical mastectomy. Although we had no formal protocol for adjuvant therapy, adjuvant chemotherapy was administered to patients with T2, T3 or lymph-node positive tumours. Two hundred and ninety-two of those patients (57.3%) received adjuvant chemotherapy including mitomycin C (given intravenously after mastectomy; total doses ranged from 20 to 40 mg for 4–6 weeks) and/or 5-fluorouracil (or its derivatives) were taken orally for 5 years. Postmenopausal patients with positive oestrogen receptors were treated with adjuvant hormone therapy from 1983. Ninety-eight patients (19.3%) received adjuvant chemotherapy and hormone therapy, and 25 patients (4.9%) received adjuvant hormone therapy. No adjuvant treatment was given to 94 patients (18.5%). The median follow-up duration of the patients was 9 years (range, 1–20).

### Pathological studies

The original histologic sections of biopsy and mastectomy specimens were reviewed. Paraffin-embedded tissue samples of 4.5 *μ*m thick sections stained with H&E were histopathologically assessed. The pathologic specimens were reviewed without any knowledge of the eventual clinical outcome. Conventional clinicopathologic features were observed and recorded, including the clinical tumour size, histological grade, LVI, and tumour necrosis (TN). The clinical tumour size was determined based on the TNM classification, the histological grade was decided based on the Bloom–Richardson grade (Bloom and Richardson, 1957), and LVI was determined based on H&E staining. Sections of the breast tumour that were stained with H&E were used to select the maximal area of all the cut surfaces of the tumour that included the invasive component.

Apoptotic cells were assessed in (H&E) stained sections. The apoptotic cells were defined as cells showing marked condensation of chromatin and cytoplasm, cytoplasmic fragments containing condensed chromatin, and intra- and extracellular chromatin fragments with a diameter of approximately 2 *μ*m (Wyllie *et al*, 1981). Apoptotic cells were counted using a standard light microscope at a 400 magnification (× 40 objective, 0.152 mm^2^ per field) in 10 randomised fields. Apoptotic index (AI) was scored as negative (<10 apoptotic cells per 10 fields) and positive (⩾10 apoptotic cells per 10 fields). The mitotic index (MI) was determined by a method described in a previous report (Baak *et al*, 1985). The nuclear membrane and the clear zone in the centre had to be absent, and clear, hairy extensions of nuclear material had to be present for them to be counted. Two parallels, and each of the two clearly separate chromosome clumps, were both counted as two mitotic figures. The number of mitotic figures was identified in 10 high-power microscopic fields (objective magnification × 40). The MI were scored as negative (<10 mitotic figures per 10 fields) and positive (⩾10 mitotic figures per 10 fields). The necrosis was divided into two categories. Coagulation necrosis in the centre area of cancer nests was defined as central necrosis, and necrosis in the intraductal component was defined as comedo necrosis (Kato *et al*, 1997).

### Immunohistochemical determinations

**Figure 1:**
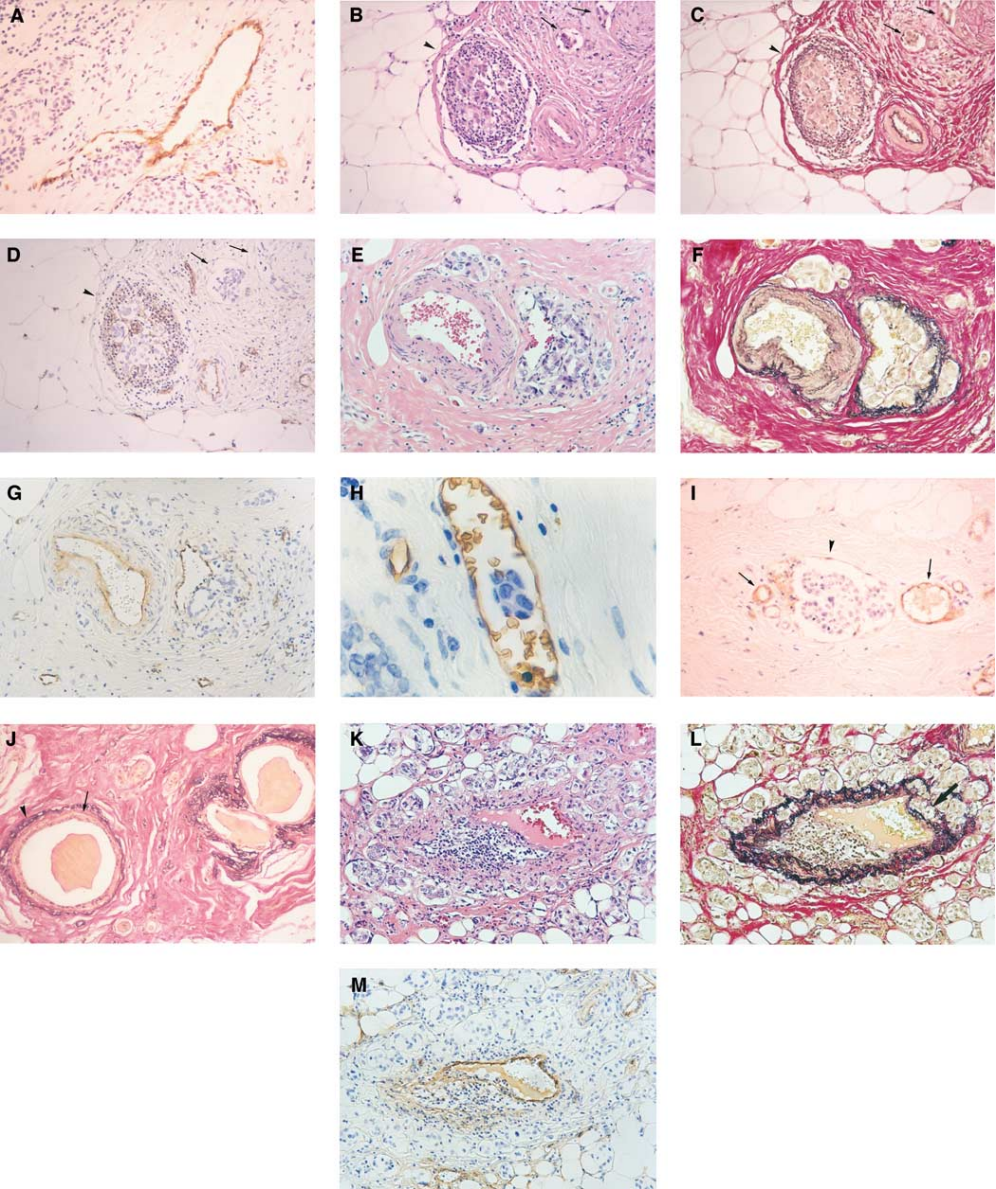
Representative examples of BVI and LVI. (1) Blood vessels directly infiltrated by cancer cells (Type I, **A**: factor VIII-related antigen staining, haematoxylin counter stain, original magnification: × 100). (2) Blood vessels filled with tumour cell emboli and lymphatic vessel invasion (Type II, **B**: H&E staining, **C**: elastica van Gieson staining, **D**: factor VIII-related antigen staining, haematoxylin counter stain, original magnification: B–D × 50). The arrowhead indicates a BVI and arrows indicate LVI with floating tumour cells. The endothelium of lymphatic vessels by factor VIII-related antigen staining was not stained. (3) Blood vessels with growth of cancer cells between endothelium and lamina elastica interna (Type III, **E**: H&E staining, **F**: elastica van Gieson staining, **G**: factor VIII-related antigen staining, haematoxylin counter stain, original magnification: E–G × 50). (4) Blood vessels with floating tumour cells (Type IV, **H**: factor VIII-related antigen staining, haematoxylin counter stain, original magnification: × 200). (5) The arrowhead indicates a lymphatic vessel with floating tumour cells and arrows indicate blood vessels. The pattern of staining in the lymphatic vessel by factor VIII-related antigen staining was very faint, discontinuous, and inconsistent in contrast to the intense and continuous staining observed in the vascular endothelium (**I**: factor VIII-related antigen staining, haematoxylin counter stain, original magnification: × 100). (6) Dilated normal ducts were identified by a zone of acidophilic non-elastic tissue inside the elastic ring surrounding the ducts. The arrowhead indicates the elastic ring and the arrow indicates acidophilic nonelastic tissue. (**J**: elastica van Gieson staining, original magnification: × 50). (7) In non-BVI, the pattern is similar to type III. There is no growth of cancer cells between the endothelium and the lamina elastica interna, while some cancer cells invaded the lamina elastica externa. The arrow indicates cancer cells invading the lamina elastica externa (**K**: HE staining, **L**: elastica van Gieson staining, **M**: factor VIII-related antigen staining, haematoxylin counter stain, original magnification: **K**–**M** × 50).

Immunostains for PCNA, p53, c-*erb*B-2, and factor VIII-related antigen were performed on the paraffin sections using the streptavidin–biotin–immunoperoxidase method as previously described (Kato *et al*, 1999). Briefly, the primary antibodies employed were an affinity-purified monoclonal anti-human-PCNA antibody (PC10, Novocastra Laboratories, Newcastle, UK) at a 1 : 100 dilution, polyclonal anti-p53 antibody (CM1, Novocastra Laboratories, UK) diluted at a 1 : 100, polyclonal anti-human c-*erb*B-2 protein antibody (Dako, Copenhagen, Denmark) at a 1 : 100 dilution, and monoclonal antibody (von Willebrand factor F8/86, Dako, Copenhagen, Denmark) applied at 1 : 200. After immunostaining, the sections were counterstained with haematoxylin. The growth fraction by PCNA staining was evaluated by counting 500 consecutive cells on one slide for each tumour with a × 400 magnification and an index of positive cells to the total number of cells (labeling index (LI)) was made. Labelling index was evaluated and scored as negative (<mean of LI) and positive (⩾mean of LI). As above, the growth fraction by p53-protein staining was evaluated by counting 500 consecutive cells and LI was made. Labelling index was evaluated and scored as negative (<mean of LI) and positive (⩾mean of LI). For c-*erb*B-2 protein expression, membrane staining in at least 50% of the tumour cells was considered positive according to the criteria of Wright *et al* (1989). Blood vessel invasion was determined using factor VIII-related antigen staining and elastica van Gieson staining as previously described (Kato *et al*, 2001,^2002^). We morphologically classified the BVI into four types according to the patterns of these blood vessels invaded by cancer cells (Figure 1

). Type I was a blood vessel directly infiltrated by tumour cells (Figure 1A), type II was a blood vessel filled with tumour cell emboli (Figure 1B–D), type III was a blood vessel with growth of cancer cells between the endothelium and the lamina elastica interna (Figure 1E–G), and type IV was a blood vessel with floating tumour cells (Figure 1H). The distinction between lymphatic vessels and blood vessels is difficult and is sometimes arbitrarily determined. While endothelial cells in the lymphatic vessels were occasionally stained by factor VIII-related antigen staining, the pattern of staining in the lymphatic vessels was very faint, discontinuous, and inconsistent in contrast to the intense and continuous staining observed in the vascular endothelium (Figure 1I). On this basis, blood vessels could be differentiated from lymphatic vessels in our study. All doubtful cases were considered to be negative. Blood vessel invasion detected by elastica van Gieson staining was defined by criteria similar to those of Weigand *et al* (1982). Blood vessels were identified by the following characteristics. Erythrocytes in the lumen, an endothelial cell lining, and the presence of elastic tissue around large vessels and ducts were identified by a zone of acidophilic nonelastic tissue inside the elastic ring surrounding the ducts (Figure 1J). When it was difficult to distinguish BVI from ductal carcinoma *in situ* (DCIS), the case was considered to be negative. All blood microvessels (capillaries and small venulae) were highlighted by staining endothelial cells with factor VIII-related antigen staining. Single brown-stained endothelial cells or clusters of endothelial cells, with or without a lumen, were counted as individual microvessels. To evaluate the best objective method to quantify microvessel density in angiogenesis, the average microvessel count (AMC) per square millimeter was employed (Kato *et al*, 1999,^2001^). One maximal area in one slide of all the cut surfaces exhibiting invasive components in each tumour was scanned at high power (× 200), and the number of microvessels in the areas along the border between cancer nests and the stroma was recorded. The average number of microvessels in all the fields scanned at high power was calculated giving the mean AMC. The segregation point of the parameter at 54.0 for AMC was deter-mined by the Cox proportional hazards regression model (Cox, 1972).

### Statistical analysis

Statistical analysis of the data was performed with the Survival Tools for Statview-J 4.5 package (Abacus Concepts, Berkeley, CA, USA) on an Apple Power Macintosh 8100/100AV. The association between each parameter was assessed by Pearson's correlation coefficient. We examined the univariate relation between prognostic indicators and 20-year RFS and OS by fitting Kaplan–Meier survival curves (Kaplan and Meier, 1958) to various levels of the prognostic indicators. We then looked for differences among the curves using the log-rank test (Mantel, 1966). The Cox proportional hazards regression model was also used for the multivariate analysis (Cox, 1972).

## RESULTS

### Clinical outcome

Among the 509 patients, there were 103 tumour-related recurrences and 83 tumour-related deaths (Table 1). In addition, there were 23 patients who were lost in the follow-up, 389 patients who are alive, and 14 patients who died of unrelated causes without recurrent tumours, with follow-up examinations of up to 20 years (median, 9 years).

### Pearson's correlation between each parameter

The rate of AMC-high and BVI-positive tumours was 158 of 485 (32.6%), 148 of 505 (29.3%), respectively. That of both AMC-high and BVI-positive tumours was 49 of 483 (10.1%) (Table 1). Table 2

**Table 2:** Pearson's correlation between each parameter

**Parameter**	**BVI**	**AMC**	**AMC/BVI**	***T***	***n***	**HG**	**LVI**	**p53**	**PCNA**	**c-*erb*B-2**	**MI**	**AI**	**Necrosis**
BVI	1												
AMC	0.032	1											
	(0.5054)												
AMC/BVI	0.459	0.897	1										
	(<0.001)	(<0.001)											
*T*	0.245	−0.069	0.045	1									
	(<0.001)	(0.1497)	(0.3463)										
*n*	0.283	0.026	0.132	0.349	1								
	(<0.001)	(0.5877)	(0.0060)	(<0.001)									
HG	0.153	−0.148	−0.065	0.319	0.164	1							
	(0.0014)	(0.0019)	(0.1776)	(<0.001)	(0.0006)								
LVI	0.261	0.083	0.183	0.208	0.422	0.048	1						
	(<0.001)	(0.0828)	(0.0001)	(<0.001)	(<0.001)	(0.3182)							
p53	0.145	−0.032	0.031	0.107	0.180	0.229	0.035	1					
	(<0.001)	(0.5073)	(0.5191)	(0.0257)	(0.0002)	(<0.001)	(0.4703)						
PCNA	0.231	0.099	0.181	0.312	0.447	0.179	0.219	0.179	1				
	(<0.001)	(0.0386)	(0.0001)	(<0.001)	(<0.001)	(0.0002)	(<0.001)	(0.0002)					
c-*erb*B-2	0.014	0.112	0.092	0.140	0.210	0.123	0.071	0.132	0.186	1			
	(0.7725)	(0.0191)	(0.0561)	(0.0036)	(0.0252)	(0.0100)	(0.1384)	(0.0060)	(<0.001)				
MI	0.184	−0.060	0.032	0.250	0.107	0.594	0.080	0.107	0.171	0.053	1		
	(0.0001)	(0.2085)	(0.5002)	(<0.001)	(0.0252)	(<0.001)	(0.0964)	(0.0257)	(0.0003)	(0.2697)			
AI	0.060	−0.014	0.019	0.090	−0.022	0.383	−0.029	0.105	0.089	0.076	0.528	1	
	(0.2138)	(0.7719)	(0.6989)	(0.0604)	(0.6464)	(<0.001)	(0.5492)	(0.0281)	(0.0625)	(0.1157)	(<0.001)		
Necrosis	0.038	−0.131	−0.096	0.140	−0.049	0.370	−0.023	0.167	0.042	0.026	0.478	0.365	1
	(0.4256)	(0.0063)	(0.0445)	(0.0035)	(0.3083)	(<0.001)	(0.6324)	(0.0005)	(0.3833)	(0.5892)	(<0.001)	(<0.001)	

BVI=blood vessel invasion; AMC=average microvessel count; *T*=clinical tumour size; *n*=lymph-node status; HG=histological grade; LVI=lymphatic vessel invasion; PCNA proliferating cell nuclear antigen; MI=mitotic index; AI=apoptotic index; *P*-values are given in parentheses.

shows the association between each parameter. When comparing BVI and clinical tumour size, lymphnode status, histological grade, LVI, p53, PCNA, and MI, there were significant correlations among them. On the other hand, AMC weakly correlated with PCNA and c-*erb*B-2, but AMC/BVI correlated with lymph-node status, LVI, and PCNA (Table 2).

### Univariate analysis

**Figure 2:**
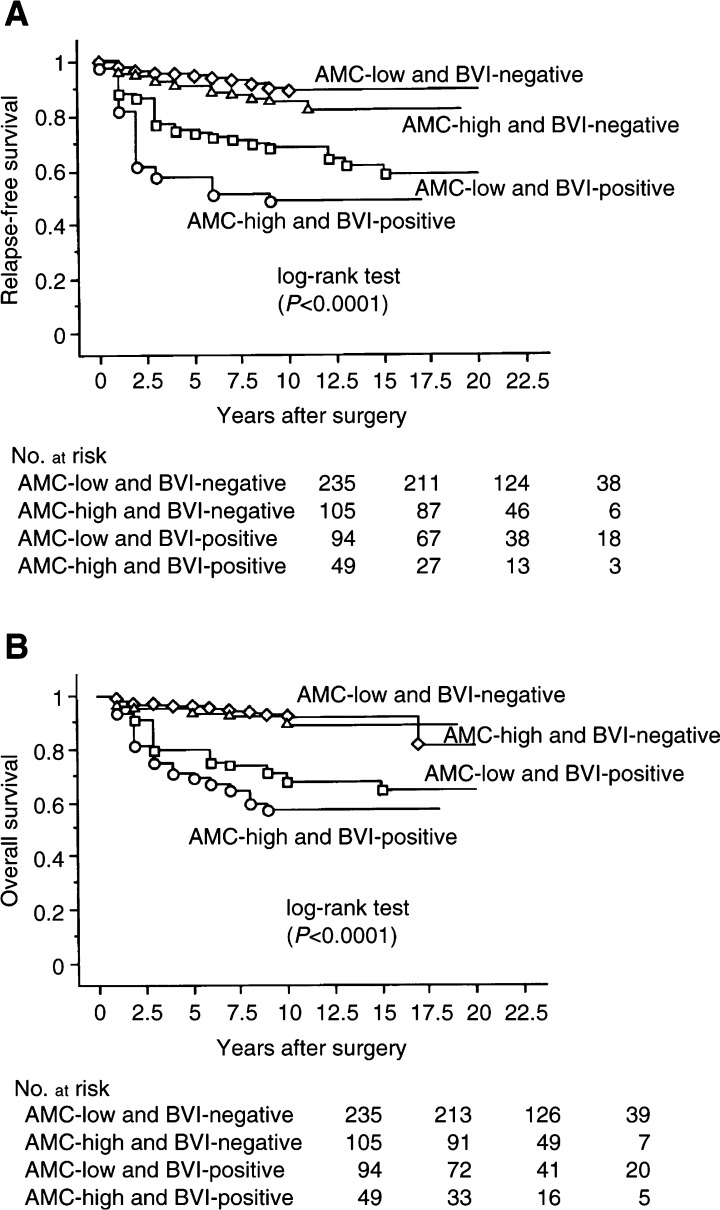
Kaplan–Meier survival curves for all patients. (**A**) RFS stratified by AMC and BVI. (B) OS related to AMC and BVI.

The prognostic factors found to be significantly associated with 20-year RFS were AMC/BVI (*P*<0.0001, Figure 2A

), clinical tumour size, lymph-node status, histological grade, LVI, p53, PCNA, MI, and TN (Table 3

**Table 3:** Univariate analysis of the value of prognostic factors for RFS and OS

	**RFS**			**OS**		
**Variable**	**RR**	**95% CI**	***P*-value**	**RR**	**95% CI**	***P*-value**
AMC						
Low *vs* high	1.7	1.1–2.5	0.0149	1.4	0.9–2.2	0.1613
BVI						
Negative *vs* positive	4.1	2.8–6.1	<0.0001	4.5	2.9–7.1	<0.0001
AMC/BVI						
Low/negative *vs* high/negative	1.7	0.9–3.1	0.1234	1.3	0.6–2.8	0.5025
Low/negative *vs* low/positive	4.0	2.3–6.8	<0.0001	4.3	2.4–7.9	<0.0001
Low/negative *vs* high/positive	7.0	4.0–12.3	<0.0001	6.3	3.3–11.9	<0.0001
Clinical tumour size (*T*)						
T1 *vs* T2	2.1	1.2–3.6	0.0062	2.1	1.1–3.9	0.0182
T1 *vs* T3	8.4	4.8–14.7	<0.0001	10.2	5.5–19.2	<0.0001
Lymph-node status						
Negative *vs* positive	5.1	3.3–7.8	<0.0001	5.8	3.5–9.7	<0.0001
Histological grade						
HG I *vs* HG II	1.9	1.1–3.2	0.0217	2.6	1.3–4.9	0.0048
HG I *vs* HG III	3.7	2.3–6.0	<0.0001	5.6	3.1–10.1	<0.0001
Lymphatic vessel invasion						
Negative *vs* positive	3.3	2.2–4.8	<0.0001	3.8	2.4–5.8	<0.0001
p53						
Negative *vs* positive	2.6	1.7–3.8	<0.0001	2.4	1.6–3.8	<0.0001
PCNA						
Negative *vs* positive	3.2	2.0–4.9	<0.0001	3.6	2.2–6.0	<0.0001
c-*erb*B-2						
Negative *vs* positive	1.4	0.9–2.2	0.0971	1.8	1.1–2.9	0.0131
MI						
Negative *vs* positive	2.4	1.6–3.5	<0.0001	2.9	1.9–4.5	<0.0001
AI						
Negative *vs* positive	1.5	0.9–2.3	0.0711	1.4	0.9–2.3	0.1491
Tumour necrosis						
None *vs* comedo	1.4	0.8–2.2	0.2050	1.5	0.9–2.6	0.1048
None *vs* central	2.2	1.3–3.5	0.0019	2.5	1.5–4.3	0.0006

RFS=relapse-free survival; OS=overall survival; RR=relative risk; AMC=average microvessel count; BVI=blood vessel invasion; PCNA=proliferating cell nuclear antigen; MI=mitotic index; AI=apoptotic index; Hazards ratio from Cox regression analysis.

). Moreover, AMC/BVI (*P*<0.0001, Figure 2B), clinical tumour size, lymph-node status, histological grade, LVI, p53, PCNA, c-*erb*B-2, MI, and TN were associated with 20-year OS. However, AI was not associated with either 20-year RFS or OS (Table 3).

### Multivariate analysis

Table 4

**Table 4:** Multivariate analysis showing independent factors of RFS and OS

**Model variable**	**1**			**2**			**3**			**4**		
	**RFS**			**RFS**			**OS**			**OS**		
	**RR**	**95% CI**	***P*-value**	**RR**	**95% CI**	***P*-value**	**RR**	**95% CI**	***P*-value**	**RR**	**95% CI**	***P*-value**
AMC												
Low *vs* high	2.3	1.5–3.5	0.0002				1.8	1.1–3.0	0.0175			
BVI												
Negative *vs* positive	2.3	1.5–3.6	0.0001				2.3	1.4–3.8	0.0011			
AMC/BVI												
Low/negative *vs* high/negative				2.3	1.2–4.6	0.0136				2.0	0.9–4.5	0.0933
Low/negative *vs* low/positive				2.3	1.3–4.1	0.0042				2.4	1.3–4.4	0.0077
Low/negative *vs* high/positive				5.5	3.0–10.1	<0.0001				4.3	2.2–8.5	<0.0001
Clinical tumour size (*T*)												
T1 *vs* T2	1.7	0.9–3.0	0.0955	1.6	0.9–2.9	0.1153	1.5	0.7–3.0	0.3025	1.4	0.7–2.9	0.3277
T1 *vs* T3	3.7	2.0–7.2	<0.0001	3.6	1.9–7.0	0.0001	3.8	1.8–7.9	0.0005	3.7	1.8–7.8	0.0006
Lymph-node status												
Negative *vs* positive	3.0	1.8–5.1	<0.0001	3.0	1.8–5.0	<0.0001	3.8	2.1–7.1	<0.0001	3.7	2.0–6.9	<0.0001
Histological grade												
HG I *vs* HG II	1.4	0.9–2.5	0.2624	1.4	0.9–2.5	0.2592	2.0	1.0–4.1	0.0476	2.1	1.0–4.2	0.0468
HG I *vs* HG III	2.1	1.3–3.7	0.0054	2.2	1.3–3.8	0.0044	3.4	1.8–6.6	0.0002	3.5	1.8–6.8	0.0002
p53												
Negative *vs* positive	1.8	1.2–2.7	0.0083	1.8	1.2–2.7	0.0066	1.5	0.9–2.4	0.0990	1.5	0.9–2.4	0.0870

RR=relative risk; RFS=relapse-free survival; OS=overall survival; 95% CI=95% confidence interval; AMC=average microvessel count; BVI=blood vessel invasion; The multivariate analysis employing Cox proportional hazard regression of prognostic factors of RFS and OS is shown. In the model regarding RFS five or six prognostic indicators are compared. In the model regarding OS five or six prognostic indicators are compared.

shows that AMC/BVI was the strongest independent prognostic factor for 20-year RFS (relative risk (RR)=5.5; *P*<0.0001) and for 20-year OS (RR=4.3; *P*<0.0001) by multivariate analysis.

### Stratification by lymph-node status

**Figure 3:**
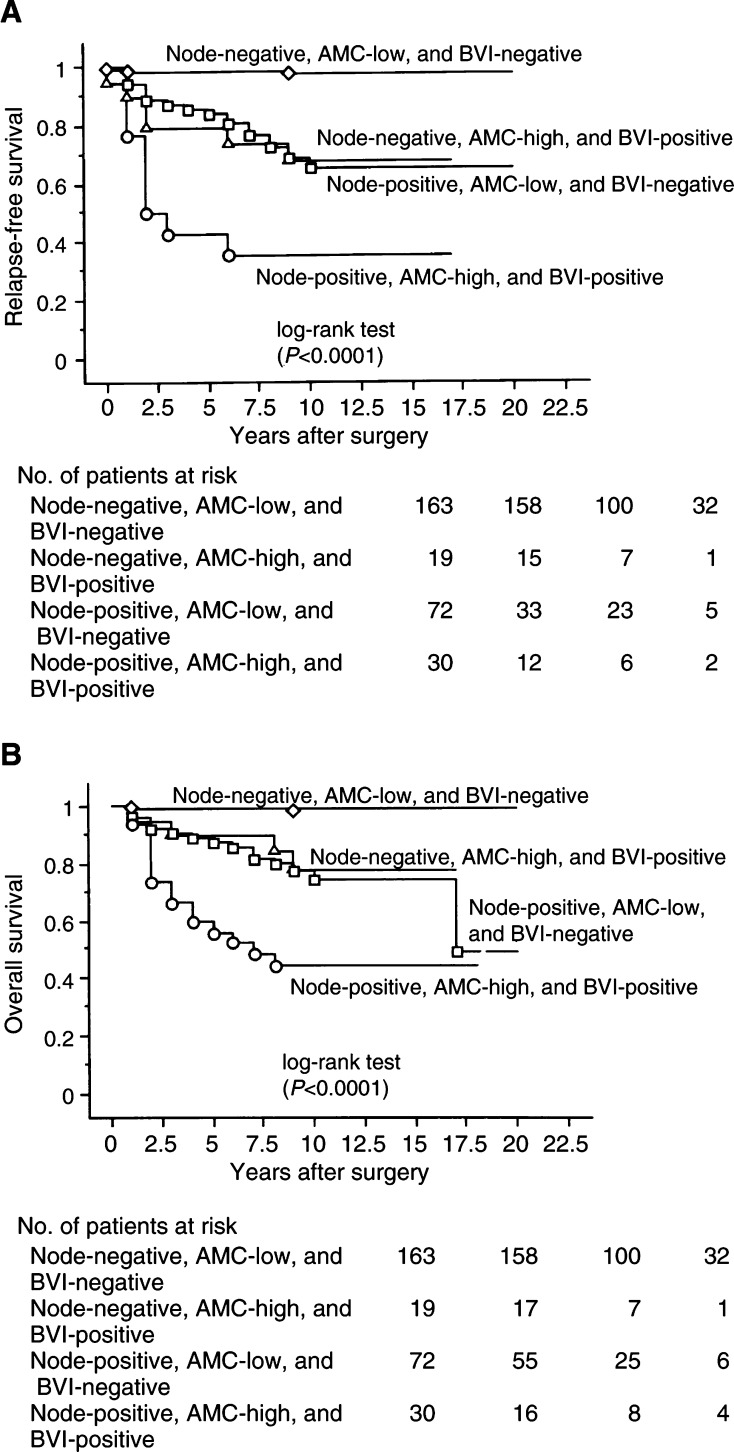
Kaplan–Meier survival curves for AMC-low and BVI-negative and AMC-high and BVI-positive patients. (**A**) RFS stratified by node, AMC, and BVI. (**B**) OS related to by node, AMC, and BVI.

When stratified by lymph-node status, a significant impact of AMC/BVI on 20-year RFS or OS was seen in patients with node-negative and -positive carcinomas by univariate analysis (*P*<0.0001 or *P*<0.0001, and *P*<0.0001 or *P*<0.0001, respectively). We fitted a model with four factors: AMC/BVI, clinical tumour size, histological grade, and p53, in patients with node-negative carcinoma, and compared them by multivariate analysis. AMC/BVI was a significant independent factor for 20-year RFS or OS (RR=19.1; 95% confident interval (95% CI) =4.6–79.5; *P*<0.0001, or RR=20.3; 95% CI=3.3–127.1; *P*=0.0013, respectively). Moreover, AMC/BVI was a significant independent factor for 20-year RFS or OS in patients with node-positive carcinoma on multivariate analysis (RR=3.4; 95% CI=1.7–6.7; *P*=0.0005, or RR=2.9; 95% CI=1.4–6.2; *P*=0.0049, respectively). Patients with node-positive carcinoma who had AMC-high and BVI-positive tumours had a higher risk of cancer-related death than the patients with node-negative carcinoma who had AMC-low and BVI-negative tumours on RFS or OS (RR=54.9; 95% CI=16.1–186.4; *P*<0.0001, RR=61.0; 95% CI=14.0–266.6; *P*<0.0001, respectively, Figures 2A and B). There was no significant difference in 20-year RFS or OS between patients with node-negative carcinoma who had AMC-high and BVI-positive tumours, and patients with node-positive carcinoma who had AMC-low and BVI-negative tumours (*P*=0.9522 and *P*=0.6867, respectively, Figures 3A and B

).

### Characteristics of the treatment

We examined the effect of treatment in all patients for RFS and OS. The modality of operation was not associated with RFS or OS. However, adjuvant chemotherapy was significantly associated with OS but not with RFS (RR=1.6; 95% CI=1.0–2.5; *P*=0.0428, or RR=1.2; 95% CI=0.8–1.8; *P*=0.4927, respectively). Using interaction terms in the Cox proportional hazards model, we examined variations in the importance of AMC/BVI across treatment subgroups. In the subgroup of 94 patients without adjuvant therapy, the relative risk of both RFS and OS also associated with AMC/BVI was significant (RR=4.7; 95% CI=1.5–14.9; *P*=0.0085, and RR=4.3; 95% CI=1.4–13.8; *P*=0.0126, respectively). On the other hand, in the subgroup of 283 patients with chemotherapy, the relative risk of both RFS and OS associated with AMC/BVI remained significantly elevated (RR=6.8; 95% CI=3.0–15.4; *P*<0.0001, and RR=6.7; 95% CI=2.6–16.8; *P*<0.0001, respectively).

## DISCUSSION

Results of the present study confirm AMC/BVI as the strongest independent prognostic factor for 20-year RFS and OS in Japanese patients with breast cancer. AMC/BVI was also a significant independent factor for 20-year RFS or OS in patients with both node-negative and positive carcinomas; especially, the effect of the presence of AMC/BVI was clearer in patients with node-negative carcinomas than in those with node-positive carcinomas.

Some clinical studies suggested that to use an antibody to CD31 may be superior to using factor VIII-related antigen (Horak *et al*, 1992; Fox *et al*, 1994), however, the other study reported that this greater sensitivity of anti-CD31 of vascular endothelium did not yield more discriminating results for predicting survival outcome than results produced with factor VIII-related antigen (Gasparini *et al*, 1994). At that point, it may lack some acuracy; however, many stromal vessels can be stained very well and be taken in to account by factor VIII-related antigen staining as we published previously (Kato *et al*, 1999). When the authors compared AMC with the highest microvessel density in one or three fields (the hot spots) in the previous study, AMC was a more reliable factor than that of the hot spots (Kato *et al*, 1999). Therefore, we used AMC in this study. The results show that AMC was an independent prognostic factor, but its prognostic impact was not as strong as lymph-node status and clinical tumour size.

The rate of BVI fell within the range of 4.2–52.0% as observed in other studies (Teel and Sommers, 1964; Friedell *et al*, 1965; Sampat *et al*, 1977; Cooperative Breast Cancer Group, 1978; Martin *et al*, 1987; Fisher *et al*, 1993; Kister *et al*, 1996; Lauria *et al*, 1996; Kato *et al*, 2002). In this study, BVI in the breast cancer patients we observed was seen in 148 cases (29.3%). On the other hand, the rate of BVI examined by Lauria *et al* (1996) was 4.2%. They detected BVI using H&E staining alone, and the prevalence of BVI was particularly low in their study. In the previous study, when we evaluated BVI by H&E staining alone, the rate of BVI presented in 6.5%, which was similar to the study by Lauria *et al* (Lauria *et al*, 1996; Kato *et al*, 2002). By H&E staining alone, it was difficult to detect blood vessels filled with tumour cell emboli, to distinguish BVI from pseudoemboli resulting from shrinkage where cancer cells peeled off from the ducts because of poor fixing, to distinguish between small blood vessel invasion and LVI, to find the blood vessels with cancer cells growing between the endothelium and the lamina elastica interna, and to distinguish BVI from DCIS covered with elastic fibre (Figure 1, Kato *et al*, 2002). Therefore, there was a possibility of overlooking some kinds of blood vessel invasion using H&E staining alone. When Weigand *et al* (1982) and others (Sampat *et al*, 1977) examined the prognostic value of BVI, BVI correlated significantly with a high rate of recurrence. The results of our study suggest that BVI is an adverse prognostic factor among patients with breast cancer as well as lymph-node status. However, when Lauria *et al* (1996) examined the prognostic value of BVI, it was associated with shorter survival by univariate analysis but not significantly an independent factor by multivariate analysis. These discrepancies may be because of some technical reasons, especially the staining methods of BVI, number of cases studied, the different follow-up period and different races.

Average microvessel count and BVI retained an independent significance in association with RFS or OS by multivariate analysis (Table 4). Independent of lymph-node status, if there were AMC-low and BVI-negative tumours, we observed similar, excellent, long-term survival. Moreover, we used both factors to define a high-risk subset of all the tumours in cases that experienced a 48.1% recurrence or a 57.4% survival probability within 17 years (Figure 1A and B). There are likely to be worse prognoses when carcinomas acquire the ability to form AMC-high and BVI-positive tumours, which then become more important indications of outcome than lymph-node status and clinical tumour size. Our study suggests that even when tumours acquire a high degree of neovascularisation, it is impossible to form distant metastases without BVI and that AMC/BVI might be the most important and strongest predictive factor for tumour haemtogenous metastasis.

In recent studies, it has been suggested that examination for bone marrow micrometastasis using immunocytochemical methods in breast cancer patients would be useful as a prognostic marker (Braun *et al*, 2000). Moreover, other investigators reported that an assessment of tumour angiogenesis and vascular invasion give a reliable indication of the likelihood of the presence of bone marrow micrometastasis in patients with breast cancer and both processes contribute to metastases (Mansi *et al*, 1991; Fox *et al*, 1997). However, as they did not distinguish BVI from LVI, the meaning of the pathway of haematogenous metastastasis remains unclear. Although some investigators have indicated it impractical to distinguish between the blood and lymphatic vessel systems as independent routes of tumour dissemination because they are so interrelated (Fisher and Fisher, 1966), the present authors examined the difference between BVI and LVI in order to distinguish the different pathways to distant metastases. As vascular invasion includes the routes of both lymphatic and haematogenous dissemination, BVI alone may show haematogenous dissemination more accurately than vascular invasion. Haematogenous metastasis can be considered a complex process of cascading interregulated sequential steps. Average microvessel count and BVI include many steps that are necessary for haematogenous dissemination, that is, they are the results of tumour invasion to extracelluar matrices and basement membranes, entering the circulatory system, and activating angiogenesis at both the primary and distant sites. Our results suggest that tumours can spread directly via the blood-vascular system from their initial growth sites, and that biologically the significance of the combination of AMC and BVI is clearly more important than that of either BVI or AMC alone.

There are some reports that indicate adequate locoregional treatment for early breast cancer may prevent secondary dissemination (Hellman and Harris, 1987; Arriagada *et al*, 1995). However, Fisher *et al* (1981) maintained that breast cancer is a systemic disease and positive lymph nodes are not important instigators of distant disease. Several investigators have found no correlation between nodal metastases and bone-marrow metastases (Harbeck *et al*, 1994; Menard *et al*, 1994). Moreover, Menard *et al* (1994) reported that pathological nodal status was not a more reliable prognostic factor than the primary tumour score, including tumour size, grading, laminin receptor, and c-*erb*B-2. Our results suggest that tumours can spread directly through the blood-vascular system from their initial growth sites, and that lymph-node status was still considered a powerful prognostic indicator; however, biologically the combination of AMC and BVI is more significant than lymph-node status for tumour haematogenous dissemination.
